# Reduction of *Atp5b* protects mice from diet-induced obesity

**DOI:** 10.1016/j.gendis.2024.101276

**Published:** 2024-03-22

**Authors:** Xiaohua Huang, Binting Qin, Zhengfeng Fang, Lianqiang Che, Yan Lin, Shengyu Xu, Yong Zhuo, Lun Hua, Xuemei Jiang, Mengmeng Sun, Hairui Wang, De Wu, Qingqiang Long, Bin Feng

**Affiliations:** a Animal Nutrition Institute, Sichuan Agricultural University, Chengdu, Sichuan 611130, China; b Key Laboratory of Animal Disease-Resistant Nutrition of Ministry of Education, Sichuan Agricultural University, Chengdu, Sichuan 611130, China; c College of Science, Sichuan Agricultural University, Ya'an, Sichuan 625014, China; d Chengdu Research Base of Giant Panda Breeding, Sichuan Key Laboratory of Conservation Biology for Endangered Wildlife, Chengdu, Sichuan 610081, China; e Guangdong Metabolic Diseases Research Center of Integrated Chinese and Western Medicine, Institute of Chinese Medicine, Guangdong Pharmaceutical University, Guangzhou, Guangdong 510006, China

Obesity related metabolic diseases, including non-alcoholic fatty liver disease, insulin resistance, hyperglycemia, and hyperlipemia, have become major chronic diseases.^1^^,^^2^ As an important enzyme for ATP production,^3^ ATP5b plays a role in many diseases, including cancer, bone homeostasis, and microvascular proliferation.^4^^,^^5^ This study was conducted to investigate the role of ATP5b in glucose and lipid metabolism. Results showed that expression of *Atp5b* was markedly reduced in diet-induced obese mice and pigs. Heterozygote *Atp5b* knockout (*Atp5b*^+/−^) mice had lower body weight, adipose tissue weight, and triglyceride content in both serum and the liver than their wild-type (WT) littermates under high-fat diet conditions. Furthermore, gene expression of *Atgl* in the liver and adipose tissue was higher in *Atp5b*^+/−^ mice than in WT mice.

The mRNA level of hepatic *Atp5b* was lower in diet-induced obese mice than in lean control mice (Fig. 1A). Expression of *ATP5b* was also decreased in the liver of diet-induced obese pigs compared with lean pigs (Fig. S1A). These data indicated that ATP5b may play a role in the regulation of hepatic glucose and lipid metabolism.

**Figure 1:**
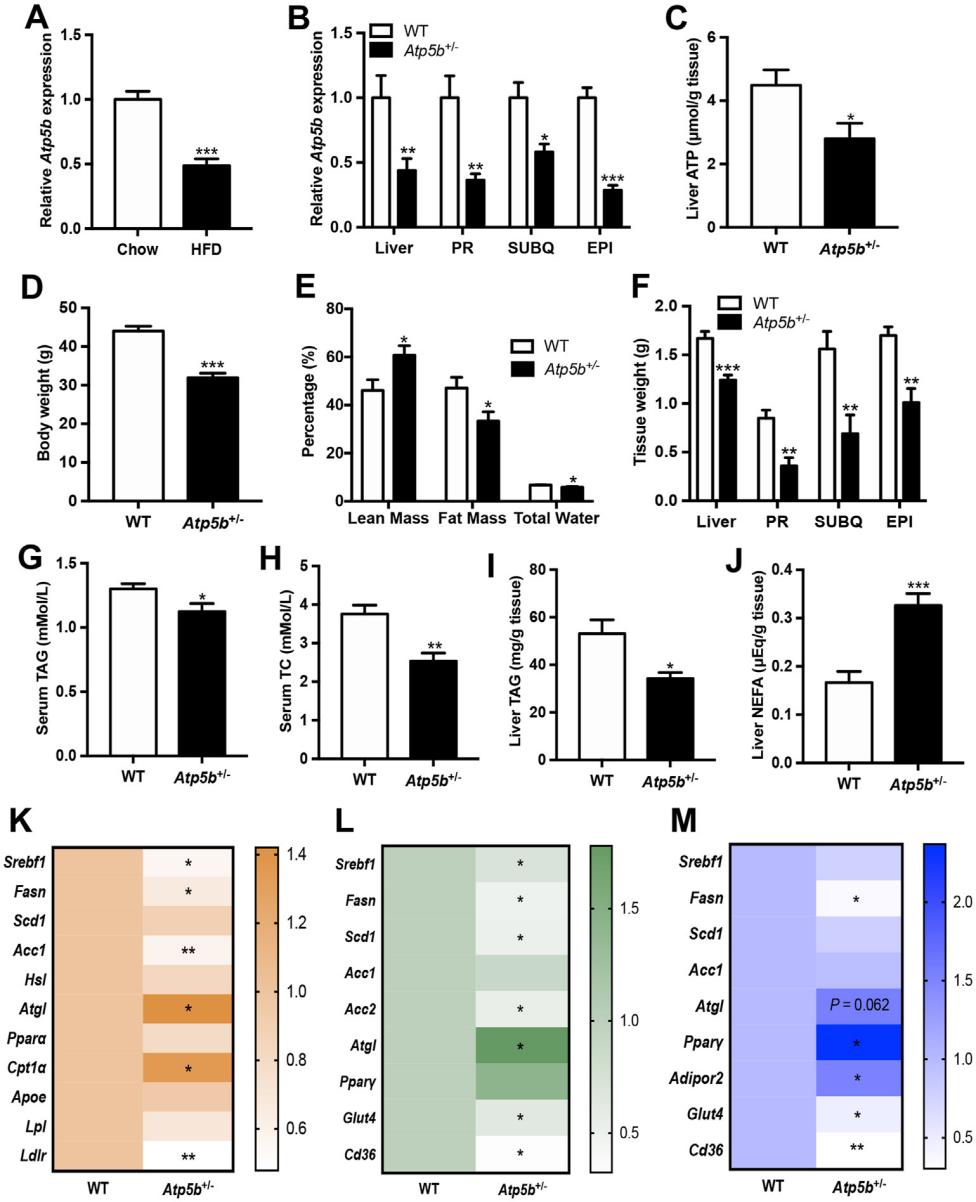
Atp5b knockdown protected mice from diet-induced obesity and hyperlipidemia. **(A)** Expression of *Atp5b* in the livers of diet-induced obesity mice and their littermate lean mice (*n* = 6 for each group). **(B–M)***Atp5b*^+/−^ mice and their wild-type littermates were fed a high-fat diet for 20 weeks and sacrificed for serum and tissue collection (*n* = 7 per group). (B) Gene expression levels of *Atp5b* in the liver, PR, SubQ, and EPI. (C) ATP content in the liver. (D) Body weight of the mice at harvest. (E) Body composition of fat and lean mass at the age of 18 weeks. (F) Tissue weight of the mice. Serum contents of TAG (G) and total cholesterol (H). TAG (I) and NEFA (J) contents in the liver. (K) Expression levels of lipid metabolic genes in the liver. (L) Expression levels of lipid metabolic genes in perirenal adipose tissue. (M) Expression levels of lipid metabolic genes in epididymal adipose tissue. The data were expressed as mean ± standard error. ∗*P* < 0.05, ∗∗*P* < 0.01, ∗∗∗*P* < 0.001 compared to control. PR, perirenal adipose tissue; SubQ, subcutaneous adipose tissue; EPI, epididymal adipose tissue; TAG, triglycerides; NEFA, non-esterified fatty acid.

We then investigated the role of ATP5b in glucose and lipid metabolism with *Atp5b* knockout mice (S–KO-01166, Cyagen, Guangzhou, China). Male *Atp5b*^+/−^ mice and WT littermates were fed a high-fat diet for 20 weeks. mRNA levels of *Atp5b* in the liver, perirenal adipose tissue, subcutaneous adipose tissue, and epididymal adipose tissue of *Atp5b*^+/−^ mice were about half of the levels in WT mice (Fig. 1B). Protein levels of ATP5b in the livers of *Atp5b*^+/−^ mice were significantly lower than those of WT mice (Fig. S1B, C). Moreover, *Atp5b* knockdown significantly decreased hepatic ATP content compared with WT control mice (Fig. 1C). Furthermore, the body weight, percentages of fat mass and total water, and tissue weight of liver and adipose tissues were much lower while the percentage of lean mass was higher in the *Atp5b*^+/−^ mice than those in WT mice (Fig. 1D–F). However, the blood glucose level, insulin tolerance test and glucose tolerance test results, and mRNA levels of hepatic gluconeogenic genes phosphoenolpyruvate carboxykinase (*Pepck1*) and glucose 6-phosphatase (*G6pc*) and their regulator peroxisome proliferator-activated receptor gamma coactivator 1 alpha (*Pgc1α*) were not changed by *Atp5b* knockdown (Figs. S1D–G). These data suggested that Atp5b might not have a regulatory effect on glucose homeostasis in obese mice.

Serum levels of triglycerides and total cholesterol were lower in *Atp5b*^+/−^ mice than in WT mice (Fig. 1G, H). Hepatic triglyceride content was also decreased while hepatic non-esterified fatty acid levels were increased by *Atp5b* knockdown compared with WT mice (Fig. 1I, J). However, serum levels of non-esterified fatty acids, low-density lipoprotein cholesterol, and high-density lipoprotein cholesterol, and liver content of total cholesterol were not changed by *Atp5b* knockdown (Figs. S1H–K). Gene expression analysis showed that *Atp5b* knockdown suppressed expression of the lipogenic genes fatty acids synthetase (*Fasn*) and acetyl-CoA carboxylase 1 (*Acc1*), and their transcriptional factor sterol regulatory element binding transcription factor 1 (*Srebf1*) and increased expression of the lipid catabolic genes adipose triglyceride lipase (*Atgl*) and carnitine palmitoyltransferase 1A (*Cpt1a*) in the liver (Fig. 1K). Of lipid transport-related genes, the mRNA level of low-density lipoprotein receptor (*Ldlr*) was decreased by *Atp5b* knockdown in the liver compared with the control (Fig. 1K). These results suggested that *Atp5b* knockdown might inhibit lipid synthesis and stimulate lipolysis in the liver, thus improving hyperlipidemia in diet-induced obese mice.

Given that the percentage of fat mass and tissue weight of fat were decreased by *Atp5b* knockdown, we investigated the expression of lipogenic and lipolytic genes in adipose tissue. Results showed that *Atp5b* knockdown suppressed expression of glucose transporter 4 (*Glut4*), fatty acid translocase 36 (*Cd36*), *Srebf1*, *Fasn*, stearoyl-CoA desaturase 1 (*Scd1*), and acetyl-CoA carboxylase 2 (*Acc2*) and increased expression of the lipolytic gene *Atgl* in perirenal adipose tissue compared with control mice (Fig. 1L). Similarly, mRNA levels of *Fasn*, *Glut4*, and *Cd36* were significantly lower while expression of adiponectin receptor 2 (*Adipor2*) and *PPARy* were higher in epididymal adipose tissue of *Atp5b*^+/−^ mice compared with WT mice (Fig. 1M). Expression of *Atgl* tended to be increased by *Atp5b* knockdown in the epididymal adipose tissue (Fig. 1M). These data indicated that *Atp5b* knockdown could suppress lipid synthesis and stimulate lipolysis in adipose tissue.

In summary, our study revealed that global reduction of *Atp5b* could protect mice from diet-induced obesity and hyperlipidemia likely by enhancing lipolysis and inhibiting *de novo* lipogenesis. Thus, ATP5b might be a therapeutic target for preventing obesity-related hyperlipidemia.

## Ethics declaration

Animal protocols were reviewed and approved by the Animal Care and Use Committee of Sichuan Agricultural University (approval number: 20210132).

## Author contributions

**B.F.**, **Q.L.**, and **X.H**. conceived and designed the experiments; **X.H.**, **B.Q.**, **Z.F.**, **L.C.**, **Y.L.**, **S.X.**, **Y.Z**, **L.H.**, **X.J.**, and **M.S**. performed the experiments; **B.F.**, **X.H.**, and **H.W**. analyzed the data; **X.H**. wrote the paper; **B.F**. and **D.W**. revised the manuscript. All authors read and approved the final manuscript.

## Conflict of interests

The authors declared no conflict of interests.

## Funding

This study was supported by the National Natural Science Foundation of China (No. 32272893) and the Program of the Giant Panda Breeding Research Foundation (China) (No. 2020CPB-C10).
