# Supplementary material for: Reduction of Atp5b protects mice from diet-induced obesity
Source: Genes Dis. 2024 Mar 22;12(2):101276. doi: 10.1016/j.gendis.2024.101276 (PMC11742349; doi:10.1016/j.gendis.2024.101276)
Supplement: Multimedia component 1 [file mmc1.docx]

**Materials and Methods**

**Animal models**

Animal protocols were reviewed and approved by the Animal Care and Use Committee of Sichuan Agricultural University. All animal studies were performed under the guide of Use and Care of Laboratory Animals. C57BL/6J wild type (WT) mice (strain No. N000013) were purchased from GenPharmatech (Nanjing, China), and *Atp5b* knockout mice (C57BL/6J background, S-KO-01166) were purchased from Cyagen biosciences (Guangzhou, China). All animals were kept in pathogen free room with 22℃ and 60% of stable temperature and humidity. *Atp5b*^+/-^ mice were mated with WT mice to generate *Atp5b*^+/-^ mice and littermate WT mice (Control). We used the heterozygote *Atp5b* knockout mice to perform the study, because the homozygous *Atp5b* knockout mice are susceptible to die. To induce obesity, mice were fed with a high-fat diet (HFD, 60% kcal from fat, D12492, Research Diet) starting at the age of 5 weeks for 20 weeks. Body composition of fat mass and lean mass was analyzed at age of 18 weeks with a body composition analyzer (AccuFat-1050, Mag-Med tech, Jiangsu, China). Mice were euthanized at age of 20 weeks after an overnight fast. Serum, liver and adipose tissues were collected for further analysis.

**Insulin tolerance test (ITT)**

After a 6-hour fasting, mice were administered with insulin (Novo, Nordisk, Beijing, China) intraperitoneally at a dosage of 1.2 Units/kg body weight (BW). Blood glucose levels were measured at 0, 15, 30, 45, 60 and 90 min after the injection of insulin, as described previously. ^1^

**Glucose tolerance test (GTT)**

Mice were intraperitoneally injected with 1.5 g/kg BW glucose (Sigma, G7021, Shanghai, China) after 12-hour fasting. Blood glucose levels were measured at 0, 15, 30, 45, 60 and 90 min after the injection, as described previously. ^2^

**Serum and liver lipid profiles**

Serum HDL-C, LDL-C, TAG, NEFA and TC levels were measured on an automatic biochemical analyzer (7020, HITACHI, Tokyo, Japan) with the respective assay kits from Kehua Bio-Engineering (Shanghai, China). Hepatic contents of TAG (Sigma, Shanghai, China), NEFA (Wako, Osaka, Japan), TC (Wako) and ATP (Elabscience, Wuhan, China) were measured according to the respective manufacturer’s instructions.

**RNA extraction and real-time PCR (qRT-PCR)**

RNA extraction and real-time PCR procedures were conducted following previously report. ^1^ Briefly, the liver tissue was subjected to total RNA extraction using TRIzol reagent (Sigma) according to the manufacturer’s instruction. The quality of the extracted total RNA was analyzed using agarose gel electrophoresis, while the concentration of RNA was determined using a spectrophotometer (NanoDrop 2000, Thermo Scientific). Complementary DNA (cDNA) synthesis was accomplished using a reverse-transcription PCR kit (RR047A, Takara, Dalian, China). Real-time PCR analysis was performed on a high-quality PCR machine (7900HT, ABI, Carlsbad, CA, USA) utilizing Power SYBR Green RT-PCR reagents (4367659, Thermo Fisher Scientific). The PCR reaction was set as a thermal cycling program of 95℃ for 10 minutes for one cycle, followed by 40 cycles of denaturation at 95℃ for 15 seconds and annealing/extension at 60℃ for 1 minute. Gene expression levels were quantified using the 2^-ΔΔCt^ method, with beta-actin serving as the reference gene. The primer sequences are showed in Table S1.

**Western blot analysis**

Protein extraction procedures were conducted following previously report. ^1^ Briefly, Liver tissue was homogenized in cell lysis buffer (Beyotime Biotechnology, Shanghai, China) supplemented with a protease inhibitor cocktail (4693116001, Roche, Mannheim, Germany) using a homogenizer. Subsequently, 30 μg of total protein was separated by SDS-PAGE polyacrylamide gel and transferred onto PVDF membranes for antibody blotting. ATP5b (17247-1-AP) and GAPDH (abs132004) antibodies were obtained from Proteintech (Wuhan, China) and Absin Biotechnology Company (Shanghai, China), respectively. After thorough washing, the membranes were incubated with suitable horseradish peroxidase-linked secondary antibodies (#7074 and #7076, Cell Signaling Technology) for 1 hour. Following additional washing steps, protein signals were detected using ECL western blotting detection reagent (1705060, BioRad) on a Molecular Imager ChemiDoc XRS+ System (BioRad).

**Statistical Analysis**

The data were analyzed using SAS 9.3 software (SAS Institute Inc., Cary, NC, USA). Firstly, the normality and homogeneity of variances of the data were assessed through univariate test. For normally distributed data, the independent t-test was employed to compare differences between two groups, while non-Gaussian and heterogeneous data were analyzed using non-parametric methods. To analyze the statistical difference of ITT and GTT, one-way repeated measures ANOVA was applied. The results were presented as mean ± SE, and statistical significance was determined at *P*-value less than 0.05.

**References**

1 Huang X, He Q, Zhu H, et al. Hepatic leptin signaling improves hyperglycemia by stimulating MAPK Phosphatase-3 protein degradation via STAT3. Cellular and Molecular Gastroenterology and Hepatology*.* 2022;14(5):983-1001.

2 Huang X, Jiang D, Zhu Y, et al. Chronic High Dose Zinc Supplementation Induces Visceral Adipose Tissue Hypertrophy without Altering Body Weight in Mice. Nutrients*.* 2017;9(10):1138.

**Supplementary Data**

**Table S1 Primers for Real-Time quantitative PCR.**

| **Genes** | **Forward** | **Reverse** |
| --- | --- | --- |
| *β-actin* | GGCTGTATTCCCCTCCATCG | CCAGTTGGTAACAATGCCATGT |
| *Atp5b* | GGTTCATCCTGCCAGAGACTA | AATCCCTCATCGAACTGGACG |
| *Glut4* | ACCGGATTCCATCCCACAAG | TCCCAACCATTGAGAAATGATGC |
| *Pparγ* | GGAAGACCACTCGCATTCCTT | TCGCACTTTGGTATTCTTGGAG |
| *Pgc1α* | TATGGAGTGACATAGAGTGTGCT | CCACTTCAATCCACCCAGAAAG |
| *Pepck1* | CGCTGGATGTCGGAAGAGG | GGCGAGTCTGTCAGTTCAATAC |
| *G6pc* | CGACTCGCTATCTCCAAGTGA | GTTGAACCAGTCTCCGACCA |
| *Fasn* | GGCTCTATGGATTACCCAAGC | CCAGTGTTCGTTCCTCGGA |
| *Scd1* | CCTACGACAAGAACATTCAATCCC | CAGGAACTCAGAAGCCCAAAGC |
| *Acc1* | CGGACCTTTGAAGATTTTGTCAGG | GCTTTATTCTGCTGGGTGAACTCTC |
| *Acc2* | GGAAGCAGGCACACATCAAGA | CGGGAGGAGTTCTGGAAGGA |
| *Srebf1* | AACTGCCCATCCACCGACTC | ATTGATAGAAGACCGGTAGCGC |
| *Adipor2* | GGAGTGTTCGTGGGCTTAGG | GCAGCTCCGGTGATATAGAGG |
| *Cpt1a* | CTCCGCCTGAGCCATGAAG | CACCAGTGATGATGCCATTCT |
| *Atgl* | CTGTGTGGAACCAAAGGACCTG | GCTACCCGTCTGCTCTTTCATC |
| *Cd36* | ATGGGCTGTGATCGGAACTG | GTCTTCCCAATAAGCATGTCTCC |


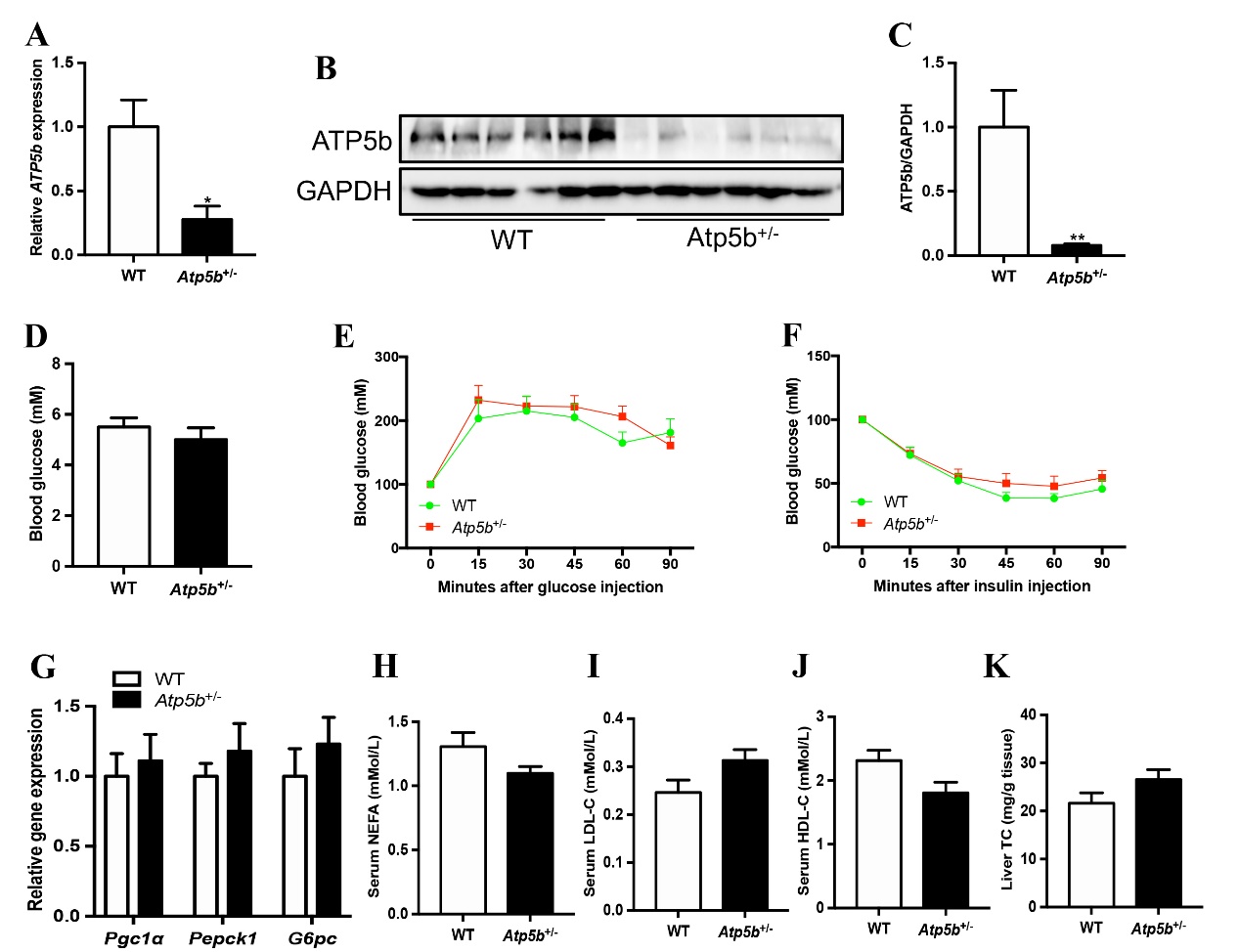


**Figure S1 The expression of ATP5b in the liver and its role in glucose and lipids metabolism.** (A) The gene expression of *ATP5b* in the liver of diet-induced obese pigs and littermate normal pigs (*N* = 6 for each group). (B-K) *Atp5b*^+/-^ mice and littermate WT mice were fed with a high-fat diet for 20 weeks. Serum and liver tissue were collected under fast condition (*N* = 7 for each group). (B,C) Protein level of ATP5b in the liver. (D) Blood glucose level at harvest. Glucose tolerance test (E) and insulin tolerance test (F) study (*N* = 7 for each group). (G) The expression level of gluconeogenic genes in the liver. (H) NEFA content in the serum. (I) LDL-C content in the serum. (J) HDL-C content in the serum. (K) TC content in the liver. Data were expressed as Mean ± SE. **P* < 0.05, ***P* < 0.01 as compared to control group.
